# A pilot study of volumetric‐modulated arc therapy for malignant pleural mesothelioma

**DOI:** 10.1120/jacmp.v17i2.5980

**Published:** 2016-03-08

**Authors:** Li Runxiao, Cao Yankun, Wang Lan

**Affiliations:** ^1^ Radiotherapy and Oncology Department Fourth Hospital of Hebei Medical University Shijiazhuang Hebei Province China

**Keywords:** pleural mesothelioma, intensity‐modulated radiotherapy/volumetric‐modulated arc therapy, plan compare; dose verification, Delta^4^ detector array

## Abstract

Malignant pleural mesothelioma (MPM) is an extremely difficult disease to treat. This pilot study investigates the feasibility of using volumetric‐modulated arc therapy (VMAT) for malignant pleural mesothelioma (MPM), and compares VMAT to static field intensity‐modulated radiotherapy (IMRT) for five patients. To identify the best treatment technique for MPM, in five patients, we made a representative comparative analysis of two kinds of techniques for radiation therapy planning: IMRT and VMAT. The plans were created for an Elekta Synergy linear accelerator with 6 MV photons using Oncentra version 4.3 treatment planning system. Dose prescription was 50 Gy to the average of the planning target volume (PTV). PTV coverage and homogeneity, dose of organs at risk, numbers of segments, MUs, and delivery time were evaluated for all techniques. VMAT allowed better homogeneous and conformity indices compared with IMRT (HI=0.17 vs. 0.12, CI=0.64 vs. 0.77, respectively, p<0.05). VMAT plan had a significantly shorter delivery time (326 s) compared with in IMRT plans (510 s), (p<0.05). In the dose verification, an average of 93.16% of the detector points passed the 3%/3 mmγ criterion for VMAT plans, while in IMRT plans the dose verification was 95.12%.(p>0.05).

PACS number(s): 87.55.D, 87.55.km, 87.56.Fc

## I. INTRODUCTION

Malignant pleural mesothelioma (MPM) is a deadly disease to treat worldwide, with the median overall survival ranging between 9 and 17 months, regardless of stage.^(1)^ In the past, surgery and chemotherapy were more commonly used for treatment of MPM than radiotherapy, due to the limitations of technique. A number of studies have investigated whether more advanced radiotherapy techniques, such as IMRT, would lead to better local control and lower doses to organs at risk (OARs) than standard RT. VMAT is a next generation of IMRT technique that can decrease treatment delivery times with similar or better plan quality for different treatment sites.^2^, ^3^, ^4^, ^5^

The aim of this study was to compare the two kinds of radiotherapy techniques and analyze the feasibility of using VMAT for MPM.

## II. MATERIALS AND METHODS

All patients underwent computed tomography (CT) simulation before radiation therapy. Simulation took place while the patients were supine and immobilized in an upper‐body cradle, with both arms overhead. The hemithorax was contoured as the clinical target volume, which included the pleural space, scars, drains sites, and involved nodal stations. This volume was then expanded to include a margin for internal motion, and an additional 0.5 to 1.0 cm planning target volume (PTV) margin was added. All PTVs and OARs were delineated by an experienced clinician. Both VMAT and IMRT plans were generated for a treatment in 25 fractions, to deliver a total dose of 50 Gy to the PTV. Both the treatment plans were optimized to cover at least 95% of the volume of PTV, and the maximal allowed point dose for spinal cord was 45 Gy, the mean dose allowed for heart was 30 Gy. All the treatment planning objectives of the plans were listed as in Table 1. VMAT and IMRT plans were generated using Oncentra version 4.3 treatment planning system (Elekta AB, Stockholm, Sweden) and commissioned for Elekta's Synergy linear accelerator with 6 MV photons. Step‐and‐shoot IMRT plans were generated using typically seven coplanar beams with a total of 50 segments. The lower limit for the segment size was $10\;{\text{cm}}^{2}$. VMAT plans were generated using dual arcs with an arc length close to the range of IMRT plan's beams. The control points within the arc were set to 4° and collimator angle was set to 0°. All IMRT and VMAT plans were generated using 6 MV photons. The conformity index $\left(\text{CI},\text{CI}=(\text{VDT}-\text{PTV}/{\text{VDT}}^{*}\text{VDT}-\text{PTV}/\text{VPTV}\right)$) and homogeneity index (HI, HI=Dmax−Dmin/Dmean) were also compared^(6)^ where DT indicates total dose of the target. We used Delta^4^ Discover detector array (ScandiDos AB, Uppsala, Sweden) to compare the dose verification, and all the data from these patients was analyzed in SPSS version 15.0 (IBM Corp., Armonk, NY).

**Table 1:** Treatment planning objectives for VMAT and IMRT plans

*Target*	*Dose—Volume Constraints*	*Weight*
PTV	Min dose 50 Gy, to 98% volume	100
	Max dose 53 Gy	100
	Uniform dose 50.5 Gy	100
*Normal Tissue*	*Dose—Volume Constraints*	*Weight*
Spinal cord	No portion may receive 45 Gy	75
Contralateral lung	$V_{5}<40\%$	10
Whole lung	$V_{5}<60\%$	15
	$V_{20}<28\%$	13
	$V_{30}<20\%$	10
Heart	$V_{30}<40\%$	10
Ring	Max dose <51 Gy	10

## III. RESULTS

**Figure 1:**
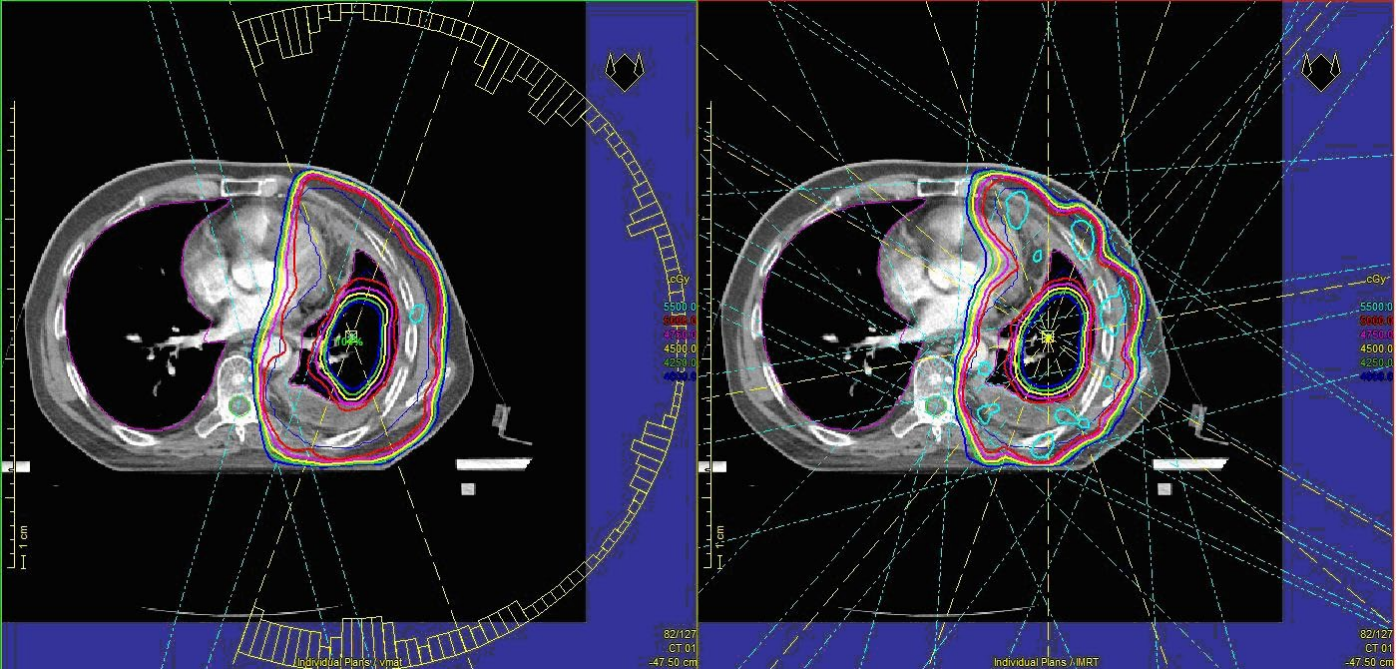
Dose distributions in transverse slice for VMAT and IMRT plans.

**Figure 2:**
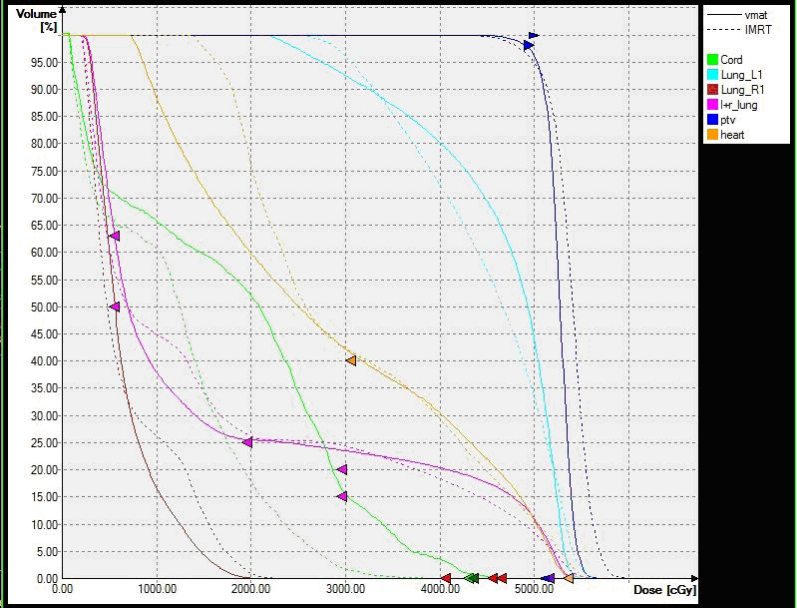
DVHs of targets and OARs for VMAT and IMRT plans.

Generally, both techniques can achieve satisfying clinical plans. VMAT plans offered tighter isodoses surfaces and encompassed the PTV with similar or better sparing of OARs. The dose distributions of IMRT and VMAT plans are shown in Fig. 1. The high dose of VMAT plans was less than IMRT plans, while the low dose was nearly the same. In addition, with VMAT plan the isodose surfaces encompassed the PTVs more smoothly and fewer hot spots outside the PTVs were observed. This means VMAT plan succeeded in producing a better dose of PTV without increasing the dose to OARs. The differences in dose and volume histogram between them are shown in Fig. 2: both $V_{110\%}$ and $V_{105\%}$ of PTV in VMAT plan decreased compared to IMRT plan, while the $D_{100},D_{98}$, and $D_{95}$ in VMAT plan increased compared to IMRT plan; ${\text{HI}}_{\text{VMAT}}=0.12,{\text{HI}}_{\text{IMRT}}=0.17,{\text{CI}}_{\text{VMAT}}=0.77,{\text{CI}}_{\text{IMRT}}=0.64$ (i.e., VMAT plans improved the conformity index and homogeneity index significantly, as shown in Table 2).

Comparison of the plan parameters for VMAT and IMRT plans generated showed significantly better sparing for most OARs (see Table 3). The maximum point dose and mean dose to spinal cord were higher with VMAT than that with IMRT. With VMAT we observed a significantly lower $V_{10},V_{20}$ (the percentage of volume receiving more than 10 Gy and 20 Gy), and average mean dose for heart and the lung. As for the $V_{5}$ of the lung, IMRT plans achieved an extremely lower dose.

Although there were more control points of VMAT plan than IMRT plan, the numbers of MUs and delivery time were decreased significantly. Dose verifications were similar between VMAT and IMRT plans (see Table 4).

**Table 2:** Dose of PTVs and significance of differences for VMAT and IMRT plans (results are averaged for five patients)

	$V_{110\%}(\%)$	$V_{105\%}(\%)$	$V_{100\%}(\%)$	$V_{95\%}(\%)$	$D_{90}(\text{Gy})$
IMRT	21.98±4.56	72.85±4.31	95.23±0.11	98.58±2.66	51.43±0.13
VMAT	7.18±4.78	55.65±3.91	95.38±1.40	99.30±2.93	51.23±0.21
*t* value	3.47	4.69	1.25	1.81	0.92
*p* value	<0.05	<0.05	>0.05	>0.05	<0.05
	$D_{\text{max}}$ (Gy)	$D_{\text{min}}$ (Gy)	$D_{\text{mean}}$ (Gy)	*HI*	*CI*
IMRT	57.28±0.44	47.98±0.45	53.66±0.21	0.17±0.02	0.64±0.39
VMAT	55.66±0.51	49.18±0.27	53.06±0.23	0.12±0.11	0.77±0.49
*t* value	2.93	4.36	2.67	5.32	8.40
*p* value	<0.05	<0.05	>0.05	<0.05	<0.05

**Table 3:** Dose of OARs and significance of differences for VMAT and IMRT plans (results are averaged for five patients)

	*Cord* $D_{\text{max}}$ *(Gy)*	*Heart* $V_{10}(\%)$	*Heart* $V_{20}(\%)$	*Heart* $V_{30}(\%)$	*Heart* $D_{\text{mean}}(\text{Gy})$
IMRT	38.85±1.53	94.98±2.57	72.59±1.36	40.67±1.15	30.09±0.46
VMAT	44.04±0.48	84.83±2.24	59.33±4.65	40.75±1.02	28.51±0.41
*t* value	3.27	4.90	10.80	0.08	4.54
*p* value	<0.05	<0.05	<0.05	>0.05	<0.05
	*Whole lung* $V_{5}(\%)$	*Whole Lung* $V_{10}(\%)$	*Whole Lung* $V_{20}(\%)$	*Whole Lung* $V_{30}(\%)$	*Whole Lung* $D_{\text{mean}}(\text{Gy})$
IMRT	58.43±3.36	43.66±1.50	25.32±1.03	21.88±0.90	16.09±0.66
VMAT	65.19±1.95	36.55±0.67	24.12±0.86	21.96±1.08	15.50±0.72
*t* value	3.52	7.88	4.33	0.17	3.42
*p* value	<0.05	<0.05	<0.05	>0.05	<0.05
	*Contralateral Lung* $V_{5}(\%)$	*Contralateral Lung* $V_{10}(\%)$	*Contralateral Lung* $V_{20}(\%)$	*Contralateral Lung* $V_{30}(\%)$	*Contralateral Lung* $D_{\text{mean}}(\text{Gy})$
IMRT	44.21±2.18	23.52±2.28	2.11±0.97	‐	7.12±0.45
VMAT	52.37±3.22	17.88±1.41	1.83±0.68	‐	5.88±0.61
*t* value	4.26	6.52	3.21	‐	3.13
*p* value	<0.05	<0.05	<0.05	‐	<0.05

**Table 4:** The parameters and significance of differences for VMAT and IMRT plans (results are averaged for five patients)

	*Total Segments*	*Total MUs*	*Delivery Time (s)*	*3 mm/3% γ*
IMRT	47±4	829±33	510±21	95.12±0.52
VMAT	118±6	657±26	326±18	93.16±0.35
*t* value	6.81	4.76	6.42	2.99
*p* value	<0.05	<0.05	<0.05	>0.05

## IV. DISCUSSION

MPM is one of the most challenging tumor entities in oncology that requires a multidisciplinary approach.^7^, ^8^, ^9^, ^10^ The large target volume that comprises the complete operated hemithorax with its complex shape and the proximity of many sensitive risk structures forces the radiation oncologist to use very sophisticated methods. Modern conformal radiation technologies, such as IMRT, open new possibilities in the treatment of complex‐shaped targets like MPM. However, Allen et al.^(11)^ point out how essential the reduction of mean lung dose in the radiotherapy of MPM is with severe pneumonitis seen after a mean dose of 15 Gy. Sterzing et al.^(12)^ compared tomotherapy and IMRT plans, and reported that, regarding the PTV, the biggest differences in plan comparison were seen in target coverage homogeneity. Tomotherapy was capable of delivering dose in excellent homogeneity. In their data, the maximum dose especially could be lowered by approximately 8 Gy on average when using tomotherapy.

Other studies have shown that the VMAT technique could achieve better plans.^13^, ^14^, ^15^, ^16^ Analysis of the data resulting from this study of VMAT for MPM shows that VMAT plans have an improved plan quality compared to IMRT plans.

IMRT can provide both dosimetric superiority and good clinical outcomes when appropriate dose constraints are used. Other studies from M.D. Anderson Cancer Center and other institutions have shown low rates of high‐grade pneumonitis and median survival times of 16 months or more with the use of IMRT. The large volume to irradiate requires a long irradiation time with IMRT techniques. Long delivery time results in patient motion during the daily treatment, with the consequence of dose delivery different from the plan that was designed in the TPS. In addition, IMRT plans need high MUs to achieve a good dose sparing, but increasing MUs means increasing probability of secondary tumors due to radiation leakage. The decreased treatment delivery time obtained with VMAT will improve patient comfort and result in a smaller impact of intrafraction movements.

It is clear that VMAT, the newest technique, is well positioned with respect to the alternative approaches from IMRT and could offer significant improvement from the logistic viewpoint. In our study, VMAT extremely reduced the delivery time and MUs, while maintaining adequate target coverage and dose sparing to the OARs. VMAT and IMRT showed both good index of PTV coverage and homogeneity. VMAT is better than IMRT.

We use the Delta^4^ detector array for dose verification. It is very important to determine the error between the calculations and effective delivery. The results show that both VMAT and IMRT plans could used for treatment with the error within the clinical limit.

## V. CONCLUSIONS

VMAT is another promising radiotherapy option for MPM. It allows reducing dose to most OARs without compromising target coverage, meanwhile keeping a shortest treatment time.

## COPYRIGHT

This work is licensed under a Creative Commons Attribution 4.0 International License.

## Supporting information

Supplementary Material Files

Supplementary Material Files
