# Supplementary material for: A pilot study of volumetric‐modulated arc therapy for malignant pleural mesothelioma
Source: J Appl Clin Med Phys. 2016 Mar 8;17(2):139–44. doi: 10.1120/jacmp.v17i2.5980 (PMC5874856; doi:10.1120/jacmp.v17i2.5980)
Supplement: Supplementary file 1 — Supplementary Material Files [file ACM2-17-139-s001.doc]

**A Pilot Study of Volumetric Modulated Arc Therapy for Malignant Pleural Mesothelioma**

*Li Runxiao, Cao Yankun，Chi Zifeng，Wang Lan，Qiu Rong，Shang Kai，Zhang Ruohui， Gao Zhe.*

*Department of Radiation Oncology，Fourth Hospital of Hebei Medical University，Shijiazhuang 050011,China*

*First author：Li Runxiao，Email：lirunxiao1984@163.com*

*Corresponding author：Cao Yankun，Email：cyk888@163.com*

**Abstract**

Malignant pleural mesothelioma (MPM) is an extremely difficult disease to treat all over the world. This pilot study presents feasibility research of volumetric modulated arc therapy (VMAT) for malignant pleural mesothelioma (MPM), and compare intensity modulated radiotherapy (IMRT) and volumetric modulated arc therapy (VMAT). In the past, the patients with MPM suffered terribly because of radiation pneumonia, as time goes by, new techniques generate new possibility. We have implemented a technique of using VMAT to report the feasibility for MPM. To identify the best treatment technique for MPM, in five patients, we made a representative comparative analysis of two kinds of techniques for radiation therapy planning, IMRT and VMAT were compared. The plans were created for an Elekta Synergy linear accelerator with 6-MV photons using Oncentra4.3 version treatment planning system. Dose prescription was 50 Gy to the average of the planning target volume (PTV). PTV coverage and homogeneity、dose of organs at risk、numbers of segments、MUs and delivery time were evaluated for all techniques. VMAT allowed better homogeneous and conformity indices compared with IMRT. (HI=0.17 VS 0.12, CI=0.64 VS 0.77, respectively, P<0.05). VMAT plan had a significantly shorter delivery time (326s) compared with in IMRT plans (510s), (P<0.05). In the dose verification, an average of 93.16% of the detector points passed the 3%/3mmγ criterion for VMAT plans, while in IMRT plans the dose verification was 95.12%.( P>0.05).

**Key Words**

Pleural Mesothelioma；Intensity-modulated radiotherapy/Volumetric modulated arc therapy； Plan Compare; Dose Verification；Delta4 detector array

**Background** Malignant pleural mesothelioma (MPM) is a deadly disease to treat, here in China, more and more patients suffered from this disease, it is also a serious problem as the worldwide. In the past, surgery and chemotherapy were used for treatment of MPM more common than the radiotherapy, the reason was the limit of radiotherapy technique, MPM is an extremely difficult disease to treat all over the world with the median overall survival ranging between 9 and 17 months, regardless of stage^[1]^. A number of studies have investigated whether more advanced radiotherapy techniques, such as IMRT, which lead to better local control and low dose of organs at risk (OARs) than standard RT. VMAT is a next generation of IMRT technique, it is supposed to decrease treatment delivery times with at least similar or even better plan quality within some other body parts ^[2-5]^.

The aim of this study was to compare the two kinds of the radiotherapy techniques and analysis the feasibility of using VMAT for MPM.

**Methods**

All patients underwent the computed tomography (CT) simulation before radiation therapy. Simulation took place while the patients were supine and immobilized in an upper body cradle, with both arms overhead. The hemi thorax was contoured as the clinical target volume, which included the pleural space, scars, drains sites, and involved nodal stations. This volume was then expanded to include a margin for internal motion, and an additional 0.5 to 1.0cm margin was added to account for patient movement (the planning target volume). All the planning target volumes (PTVs) and organs at risk (OARs) were delineated by experienced clinician. Both VMAT and IMRT plans were generated for a treatment in 25 fractions, to deliver a total dose of 50Gy to the PTV. Both the treatment plans were optimized to cover at least 95% of the volume of PTV, and the maximal allowed point dose for spinal cord was 45Gy, the mean dose allowed for heart was 30Gy. VMAT and IMRT plans were generated using Oncentra4.3 version treatment planning system and commissioned for Elekta Synergy linear accelerator with 6-MV photons. Step-and-shoot IMRT plans were generated using typically 7 coplanar beams with a total of 50 segments. The lower limit for the segment size was 10 cm^2^. VMAT plans were generated using dual arcs with an arc length close to the range of IMRT plan’s beams, the control points within the arc was set to 4^。^and collimator angle was set to 0^。^. All IMRT and VMAT plans were generated using 6MV photons. All the objectives of optimization should be adapted during the process to achieve better plan quality. Beside the dose of PTV and OARs, conformity index (CI, CI= (V_DT-PTV_/V_DT_*V_DT-PTV_/V_PTV_)) and homogeneity index (HI, HI=D_max_—D_min_/D_mean_) were also computed to comparison We used Delt4 detector array to compare the dose verification, and all the data from these patients was analyzed in SPSS 15.0 version.

**Results**

Generally, both techniques can achieve satisfying plans for clinical. VMAT plan offered tighter isodose surfaces encompassed the PTV with similar or better sparing of lower dose of OARs, dose distributions of IMRT and VMAT plans are shown in Figure1:the high dose of VMAT plans was less than IMRT plans, while the low dose of them were nearly the same. In addition, with VMAT plan the isodose surfaces encompassed the PTVs more smoothly and fewer hot spots outside the PTVs were observed. This means VMAT plan succeeded in producing a better dose of PTV without dose increasing of OARs. The differences in dose and volume histogram between them are shown in Figure2: both V_110%_ and V_105%_ of PTV in VMAT plan were depressed to IMRT plan, while the D_100_、D_98_ and D_95_ in VMAT plan were increased to IMRT plan; HI_VMAT_=0.12, HI_IMRT_=0.17, CI_VMAT_=0.77, CI_IMRT_=0.64, i.e. VMAT plans improved the conformity index and homogeneity index significantly, these were shown in Table 1.

Figure 1 Dose distributions in transverse slice for VMAT and IMRT plans

**
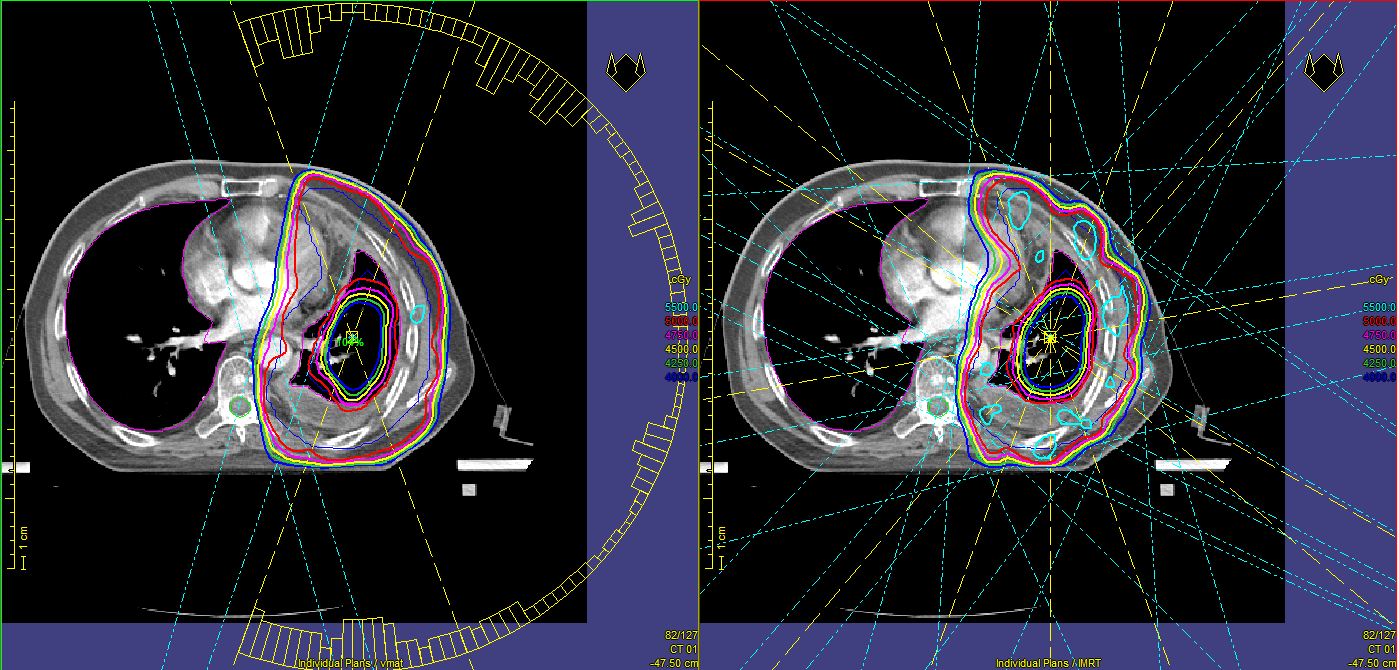
**

Figure 2 DVHs of targets and OARs for VMAT and IMRT plans


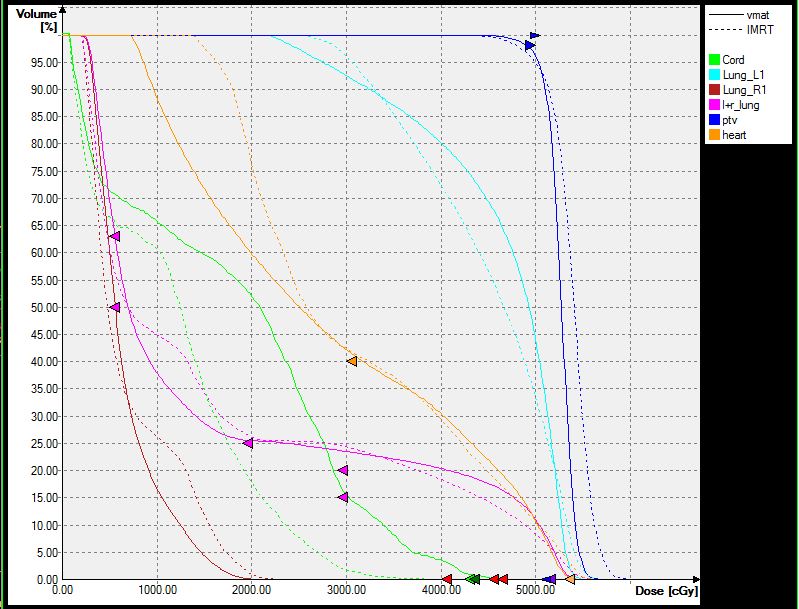


Table 1 Dose of PTVs and significance of differences for VMAT and IMRT plans

|  | V_110%_ (%) | V_105%_ (%) | V_100%_ (%) | V_95%_ (%) | D_90_ (Gy) |
| --- | --- | --- | --- | --- | --- |
| IMRT | 21.98±4.56 | 72.85±4.31 | 95.23±0.11 | 98.58±2.66 | 51.43±0.13 |
| VMAT | 7.18±4.78 | 55.65±3.91 | 95.38±1.40 | 99.30±2.93 | 51.23±0.21 |
| *t* value | 3.47 | 4.69 | 1.25 | 1.81 | 0.92 |
| P value | <0.05 | <0.05 | >0.05 | >0.05 | <0.05 |
|  | D_max_ (Gy) | D_min_ (Gy) | D_mean_ (Gy) | HI | CI |
| IMRT | 57.28±0.44 | 47.98±0.45 | 53.66±0.21 | 0.17±0.02 | 0.64±0.39 |
| VMAT | 55.66±0.51 | 49.18±0.27 | 53.06±0.23 | 0.12±0.11 | 0.77±0.49 |
| *t* value | 2.93 | 4.36 | 2.67 | 5.32 | 8.40 |
| P value | <0.05 | <0.05 | >0.05 | <0.05 | <0.05 |

Comparison of the plan parameters for VMAT and IMRT plans generated showed significantly better sparing for most OARs (see Table 2). The maximum point dose and mean dose to spinal cord were higher with VMAT than that with IMRT. With VMAT we observed a significantly lower V_10_、V_20_(the percentage of volume receiving more than 10 Gy and 20Gy) and average mean dose for heart and the lung; As for the V_5_ of the lung, IMRT plans achieved extremely lower dose.

**、**

Table 2 Dose of OARs and significance of differences for VMAT and IMRT plans

|  | Cord  D_max_ (Gy) | Heart  V_10_ (%) | Heart  V_20_ (%) | Heart  V_30_ (%) | Heart  D_mean_ (Gy) |
| --- | --- | --- | --- | --- | --- |
| IMRT | 38.85±1.53 | 94.98±2.57 | 72.59±1.36 | 40.67±1.15 | 30.09±0.46 |
| VMAT | 44.04±0.48 | 84.83±2.24 | 59.33±4.65 | 40.75±1.02 | 28.51±0.41 |
| *t* value | 3.27 | 4.90 | 10.80 | 0.08 | 4.54 |
| P value | <0.05 | <0.05 | <0.05 | >0.05 | <0.05 |
|  | lung  V_5_ (%) | lung  V_10_ (%) | lung  V_20_ (%) | lung  V_30_ (%) | lung  D_mean_ (Gy) |
| IMRT | 58.43±3.36 | 43.66±1.50 | 25.32±1.03 | 21.88±0.90 | 16.09±0.66 |
| VMAT | 65.19±1.95 | 36.55±0.67 | 24.12±0.86 | 21.96±1.08 | 15.50±0.72 |
| *t* value | 3.52 | 7.88 | 4.33 | 0.17 | 3.42 |
| P value | <0.05 | <0.05 | <0.05 | >0.05 | <0.05 |

Although there were more control points of VMAT plan than IMRT plan, the numbers of MUs and delivery time were decreased significantly. Dose verifications were similar between VMAT and IMRT plans (see table 3).

Table 3 The parameters and significance of differences for VMAT and IMRT plans

|  | Total segments | Total  MUs | Delivery time(s) | 3mm/3%  γ |
| --- | --- | --- | --- | --- |
| IMRT | 47±4 | 829±33 | 510±21 | 95.12±0.52 |
| VMAT | 118±6 | 657±26 | 326±18 | 93.16±0.35 |
| *t* value | 6.81 | 4.76 | 6.42 | 2.99 |
| P value | <0.05 | <0.05 | <0.05 | >0.05 |

**Discussion**

MPM is one of the most challenging tumor entities in oncology that requires a multidisciplinary approach ^[6-9]^. The big target volume that comprises the complete operated hemi thorax with its complex shape and the proximity of many sensitive risk structures forces the radiation oncologist to use very sophisticated methods Modern conformal radiation technologies as IMRT open new possibilities in the treatment of complexly shaped targets as MPM. However, Allen et al. point out how essential the reduction of mean lung dose in the radiotherapy of MPM is with severe pneumonitis seen after a mean dose of 15Gy ^[10]^. Florian Sterzing et al. compared tomotherapy and IMRT plans, and reported that regarding the PTV the biggest differences in plan comparison were seen in target coverage and homogeneity, tomotherapy was capable of delivering dose in excellent homogeneity. In their data especially the maximum dose could be lowered by approximately 8 Gy in average when using tomotherapy ^[11]^.

Here is the common view that VMAT technique could achieve better plans ^[12-15]^ , analysis of the data resulting from this study of VMAT for MPM shows that VMAT plans have an improved plan quality compared to IMRT plans.

IMRT can provide both dosimetric superiority and good clinical outcomes when appropriate dose constraints are used. Other studies from M.D. Anderson Cancer Center and other institutions have shown low rates of high-grade pneumonitis and median survival times of 16 months or more with the use of IMRT. The large volume to irradiate requires a long irradiation time with IMRT techniques. Long delivery time result in patients motion during the daily treatment, with the consequence of dose delivery different from the plan that designed in the TPS. Beside that, IMRT plans need high MUs to achieve a good dose sparing, but increasing MUs means increasing probability of secondary tumors due to radiation leakage. The decreased treatment delivery time obtained with VMAT will improve patient comfort and result in a smaller impact of intra-fraction movements.

It is clear that VMAT , the newest technique is well positioned with respect to the alternative approaches from the IMRT and could offer significant improvement from. the logistic viewpoint. VMAT had extremely reduced the delivery time and MUs, while maintaining adequate target coverage and dose sparing to the OARs. VMAT and IMRT showed both good index of PTV coverage and homogeneity. VMAT is better than IMRT.

We use the Delta4 detector array to the dose verification. It is very important to make sure the error between the calculations and effective delivery. The result showed, both VMAT and IMRT plans could used for treatment with the error within the clinical limit.

**C****onclusions**

VMAT is considered to be the first treatment option for this case. It allows reducing dose in most OARs without compromising target coverage, meanwhile keeping a shortest treatment time.

**References**

[1]Anne S.Tsao, Ignacio Wistuba, Jack A. Roth, et al. Malignant Pleural Mesothelioma. J Clin Oncol,2009,27:2081-2090.

[2]Bedford JL:Treatment planning for volumetric modulated arc therapy. Med Phys, 2009, 36:5128-5138.

[3]Verbakel WF, Cuijpers JP, Hoffmans D, Bieker M, Slotman BJ, Senan S: Volumetric intensity-modulated arc therapy vs. conventional IMRT in head-and-neck cancer: a comparative planning and dosimetric study. Int J Radiat Oncol Biol Phys 2009, 74:252–259.

[4] Rao M, Yang W, Chen F, Sheng K, Ye J, Mehta V, Shepard D, Cao D: Comparison of Elekta VMAT with helical tomotherapy and fixed field IMRT: plan quality, delivery efficiency and accuracy. Med Phys 2010, 95:142–148.

[5] Lu SH, Cheng JC, Kuo SH, Lee JJ, Chen LH, Wu JK, Chen YH, Chen WY, Wen SY, Chong FC, Wu CJ, Wang CW: Volumetric modulated arc therapy for nasopharyngeal carcinoma: A dosimetric comparison with TomoTherapy and step-and-shoot IMRT. Radiotherapy Oncol 2012, 104:324-330.

[6]Neragi-Miandoab S. Multimodality approach in management of malignant pleural mesothelioma. Eur J Cardiothoracic Surg 2006;29:14–9.

[7] Sterman DH, Albelda SM. Advances in the diagnosis, evaluation, and management of malignant pleural mesothelioma. Respirology 2005;10:266–83.

[8] UngYC, Yu E, Falkson C, et al. Therole of radiation therapy in malignant pleural mesothelioma: a systematic review. Radiother Oncol 2006.

[9] Yajnik S, Rosenzweig KE, Mychalczak B, et al. Hemithoracic radiation after extrapleural pneumonectomy for malignant pleural mesothelioma. Int J Radiat Oncol Biol Phys 2003;56:1319–26.

[10] Allen AM, Czerminska M, Janne PA, et al. Fatal pneumonitis associated with intensity-modulated radiation therapy for mesothelioma. Int J Radiat Oncol Biol Phys 2006;65:640–5.

[11]Florian Sterzing, Gabrele Sroka-perez, Kai Schubert, et al. Evaluating target coverage and normal tissue sparing in the adjuvant radiotherapy of malignant pleural mesothelioma helical tomotherapy compared with step-and-shoot imrt. Radiotherapy Oncol 2008, 86:251-257.

[12]Otto K. Volumetric modulated arc therapy: IMRT in a sigle gantry arc. Med Phys, 2008, 35:310-317.

[13] Yoo S, Wu QJ, Lee WR, et al. Radiotherapy treatment plans with VMAT for prostate cancer involving seminal vesicles and lymph nodes. Int J Radiation Oncology Biology Phys, 2010, 76:935-942.

[14]Yu CX．Intensity modulated arc therapy with dynamic multi leaf collimation：alternative to tomotherapy．Phys Med Biol，1995，40：1435-1449．

[15] Guckenberger M, Richler A, Krirger T, et al. Is a single arc sufficient in volumetric modulated arc therapy (VMAT) for complex shaped target volumes? Radiation Oncol, 2009, 93:259-265.
